# A Novel Byte-Substitution Architecture for the AES Cryptosystem

**DOI:** 10.1371/journal.pone.0138457

**Published:** 2015-10-22

**Authors:** Fakir Sharif Hossain, Md. Liakot Ali

**Affiliations:** 1 Department of Electrical and Electronic Engineering, International Islamic University Chittagong, Chittagong, Bangladesh; 2 Institute of Information and Communication Technology, Bangladesh University of Engineering and technology, Dhaka, Bangladesh; University of Kent, UNITED KINGDOM

## Abstract

The performance of Advanced Encryption Standard (AES) mainly depends on speed, area and power. The S-box represents an important factor that affects the performance of AES on each of these factors. A number of techniques have been presented in the literature, which have attempted to improve the performance of the S-box byte-substitution. This paper proposes a new S-box architecture, defining it as ultra low power, robustly parallel and highly efficient in terms of area. The architecture is discussed for both CMOS and FPGA platforms, and the pipelined architecture of the proposed S-box is presented for further time savings and higher throughput along with higher hardware resources utilization. A performance analysis and comparison of the proposed architecture is also conducted with those achieved by the existing techniques. The results of the comparison verify the outperformance of the proposed architecture in terms of power, delay and size.

## Introduction

Encryption algorithms are broadly classified as symmetric and asymmetric algorithms based on the type of keys used. Due to the complexity of asymmetric algorithms, symmetric ciphers are always preferred for their speed and simplicity. AES (the Rijndael algorithm) is one such symmetric algorithm for encryption which replaced triple-DES and eventually became the number one choice for security algorithms all over the world by 2001. Therefore, NIST declared AES to be the standard for privacy and security of information [1]. Since then, it has been used for countless different applications ranging in size and scale such as military, e-banking and different data communication purposes.

There have been many novel design techniques for AES that focus on obtaining high throughput or low area usage. The demand for fast and area-efficient AES implementations is rapidly growing and is becoming more crucial than the demand for a smaller active device on the chip. The traditional basic lookup table implementations are relatively fast and can achieve better performance with some modifications. The use of smaller look up tables (LUTs) of different sizes ranging from 16 to 128 bytes has become more reliable for getting higher speed. This paper proposes the LUT of small size, which reduces the indexing and provides satisfactory results in terms of power, area and speed. A significant portion of the overall silicon area for implementing AES architectures is occupied by the S-box. The size of SubBytes is, in turn, determined by the number of S-boxes and their concrete implementation. Various implementation options for the AES S-box have been investigated in the recent past [2–12]. One of our previous work [13], we show that the speed of the AES processor can be maximized by optimizing the S-box and MixColumn stages. That work reports the high performance in terms of throughput and latency. In recent years, hardware implementations in CMOS technology received a lot of preference due to their good performance. An initial attempt of optimizing AES S-box is introducing the composite field decomposition technique of S-box, in which a multi-stage positive polarity Reed-Muller architecture has been introduced [14]. In this S-box, the hazard-transparent XOR gates are located after the other gates which may block the hazards.

Moreover, there are a lot of applications coming out at present, such as contactless smart card, wireless sensor network, small computing devices etc., where low power is important. This is the reason why a number of research works have been proposed and further research works are still continuing focusing on low power [15]. The use of embedded functional blocks instead of general purpose logic elements is a good idea to reduce the dynamic power consumption of the designs [16]. It is seen that internal routing of embedded system block is more power efficient than the routing used for general purpose logic. It has been also commented that a large amount of power consumption in an AES processor is due to its S-box and that it is responsible for consuming 75% of its total power. Clock gating is another commonly used technique for dynamic power reduction [17]. Studies show that basic pipelining technique can reduce 31% dynamic power consumption, while it can increase up to 82% due to heavy pipelining. Wong [18] aims to have achieved a high throughput compact AES S-box with minimal power consumption. They have proposed a novel pipelining arrangement over the compact composite field S-box such that both high throughput and low power are optimized.

Some literatures provided good results for FPGA implementations too. The optimized implementation on composite field arithmetic has introduced to reduce both static and dynamic power consumption of S-box along with pipelining and dynamic voltage scaling [19]. Besides, minimizing the supply voltage apparently reduces the power dissipation in designs. The T-box AES design is intended to have high throughput and low power usage [20]. The T-box method has its potential in embedded system to have power and energy efficient design since it relies on embedded RAM blocks rather than general purpose logic. Another technique is to use low data path width for AES design in order to reduce the power consumption [21].

Now-a-days there are a lot of applications coming in the market where an increasing number of battery-powered embedded systems like PDAs, cell phones, networked sensors, smart cards, RFID etc. are used to store, access, manipulate, or communicate sensitive data. Eventually, this makes security a very important concern. Since these devices are resource constrained and battery powered, low power and small area are some of the primary requirements. This paper focuses on the solution of this particular problem and has presented a novel technique in designing a low power, least delay and area efficient S-box for an AES processor. In the process of proving the claim, a fair comparison among area, delay and power estimation is presented based on target delay. The graphical representation of (i) GE versus target value for critical path delay, (ii) Total Power versus target value for critical path delay and (iii) Power area product versus target value for critical path delay are performed which shows the novelty of the work.

The remainder of this paper is organized as follows. In Section 2 The AES S-box and its different implementation techniques along with the realization are discussed. In Section 3, the S-box algorithm is proposed and followed by the implementation of SubByte in both CMOS and FPGA is discussed in Section 4. Furthermore, Section 5 presents the results and performance analysis of proposed S-box architecture followed by comparison to other recent related works in the Section 6. We conclude in Section 7.

## AES S-box and Related Implementation Strategies

It is well known that the S-box is the most weighted transformation among the four rounds of the AES algorithm. The substitution byte (S-box) serves the purpose of bringing confusion to the data that is to be encrypted. The S-box is a 16 by 16 matrix box containing a total of 256 byte hexadecimal and indexed in a row and column pattern. The S-boxes used in the SubBytes function are created in such a way that they are invertible for using as inverse S-boxes in the InvSubBytes function. The S-box computation involves basically two steps, the multiplicative inverse and the affine transformation. The multiplicative inverse is complex to perform in GF (2^8^), so in order to simplify, composite field arithmetic is used by some researchers. This sort of implementation has good response in terms of area, but due to the large signal activities, consumes more power. In order to achieve high throughput and low power, many literatures present the hardware look-up table implementation of S-box. This paper presents an optimized look-up table implementation of S-box.

Logically, the SubBytes transformation substitutes all of the 16 bytes of the state independently using the S-box. In software, the S-box is typically realized in the form of a look-up table since inversion in the Galios Field (GF) cannot be calculated efficiently on general-purpose processors. In case of hardware, on the other hand, the implementation of the S-box is directed to the desired trade-off among area, delay, and power consumption. The most obvious implementation approach of S-box takes the form of hardware look-up tables. Our proposed design will explain how the hardware look-up table works efficiently in the next couple of sections. More sophisticated approaches include the calculation of S-box function in hardware using its algebraic properties [22]. Composite field based design is a good example of calculating S-box. The main drawback of composite field approach is greater power consumption, but delay is much less compared to other architectures. The minimal power consumption for implementing the AES S-box is approached by Bertoni et al [23]. He used an intermediate one-hot encoding of the input and arbitrary logic functions (including cryptographic S-boxes) to realize minimal power consumption. Relatively large silicon area is the main drawback of this approach. Tiltech [24] describes a total of eight different implementations of the AES S-box in which he grouped them into three basic categories: look-up implementations, calculating implementations, and low-power implementations. In order to validate our approach, a fair comparison with Tiltech’s work will be established, as he covers all the implementations aspects of S-box.

On the other hand, Implementations which calculate the S-box transformation in hardware were first proposed by Wolkerstorfer et al. [25] and Satoh et al. [26]. The former approach decomposes the elements of finite field into polynomials over the subfield and performs inversion there. Canright [27] improved the calculation of the S-box by switching the representation to a normal basis. The low-power approach of Bertoni et al. use a decode stage to convert the eight bits of the input byte and the control bit which selects encryption or decryption into a one-hot representation consisting of 29 ¼ 512 bits. Due to the decoder-permute-encoder structure, there is only very little signal activity within the circuit when the input changes, resulting in low power consumption. It should be noted that the structure of Bertoni’s approach makes it easily possible to introduce pipeline stages. However, it may be necessary to add a large number of additional flip-flops when the pipeline stage is placed between the decoder and encoder. It results large power consumption. This proposed algorithm substitutes a byte through small table look-up without inserting any flip flop when pipelined. Therefore, a change of a few input bits affects the evaluation of all output bits separately. As normally some output bits will remain unchanged, the signal activity within this particular path is low. Thus it limits the overall power consumption of the S-box. The second implementation of Bertoni uses a two stages decoder structure so as to reduce the critical path delay of the circuit. This paper approaches a single stage decoder function which performs better compared to Bertoni.

Elazm [28] shows a composite Galois Field design of S-box to reduce the size and the delay of the circuit. Transmission gate is employed to reduce power consumption of the mentioned circuit. This design suffers long critical path delay due to switching and glitch. The proposed work ignores the algebraic properties of the substitution and simply implements the Boolean equations of the input/output relation. Therefore, less switching activities ensure lower power consumption. Due to simple Boolean implementations, the synthesizer has a much higher degree of freedom for optimizing the proposed circuit, which allows for a shorter critical path at a little expense of the silicon area.

In a recent paper, Shanthini [29] presents an optimized composite field arithmetic S-box implementation in a four stage pipeline. Here the S-box operation is divided into the Galios Field multiplication and its inverse operation and later illustrated in a step-by-step manner. The main constrain is appeared when considered the critical path versus the area-power product. Comparatively, the implementation of our proposed work on FPGA had a very good result in terms of area, power and product. Another work by Ahmed [30] presents a full custom CMOS design of S-box/Inversion S-box (Inv S-box) mapped in low power GF (2^8^) inversion. He used polynomial basis using composite field arithmetic and got a fascinating result in both silicon area and power consumption. But the main drawback is its critical path delay, which is five to six times than that of the proposed design. The next Section shows the proposed S-box architecture in detail.

## Proposed S-box Architecture

In the previous Section, the three general techniques for realizing the S-box has already been discussed, of which, the proposed architecture uses the combination of both the Hardware and the Software technique. In one case the multiplicative inverse in GF (2^8^) is realized as look-up table, while the affine transformation is computed as in hardware techniques [24]. This approach has the benefits of avoiding the complexity of inversion and reducing LUT space requirements to half that of the LUT used for the whole S-box. The basic idea of this approach is that the original S-box is broken down into a set of smaller size multiplexer-switched truth-table of say n-variable functions using the Shannon expression. The smaller tables are then mapped into n-LUT of Altera FPGA. The mapping of LUTs is provided by the following pseudo code:

INITIALIZING, i, j, GR

GR ← 3 (Binary value)

CASE GR OF

CASE(00): for i ← 0 TO 3 for j ← 0 TO 7

DO (i,j) ← (i,j) AND DO (i+1, j) ← (i, j+8)

ENDFOR

CASE(01): for i ← 4 To 7 for j ← 0 To 7

DO (i,j) ← (i,j) AND DO (i+1, j) ← (i, j+8)

ENDFOR

CASE(10): for i ← 8 TO B for j ← 0 TO 7

DO (i,j) ← (i,j) AND DO (i+1, j) ← (i, j+8)

ENDFOR

CASE(11): for i ← C TO F for j ← 0 TO 7

DO (i,j) ← (i,j) AND DO (i+1, j) ← (i, j+8)

ENDFOR

ENDCASE

Initially, the single S-box is decomposed into 4 tables of 64 bytes, which are called as groups. Within a group, there are 16 separate tables of 2×2 bytes. The decomposition of these tables is similar to the group formation itself. In case of a single S-box of 16×16 bytes hexadecimal values, the substitution of a single byte of AES state from the S-box requires a 16×16 table look up. Therefore, large number of iterations are initiated which results longer delay and larger power consumption. On the contrary, the proposed design constructs an effectively small look-up table of only 2×2 bytes.

**Fig 1:**
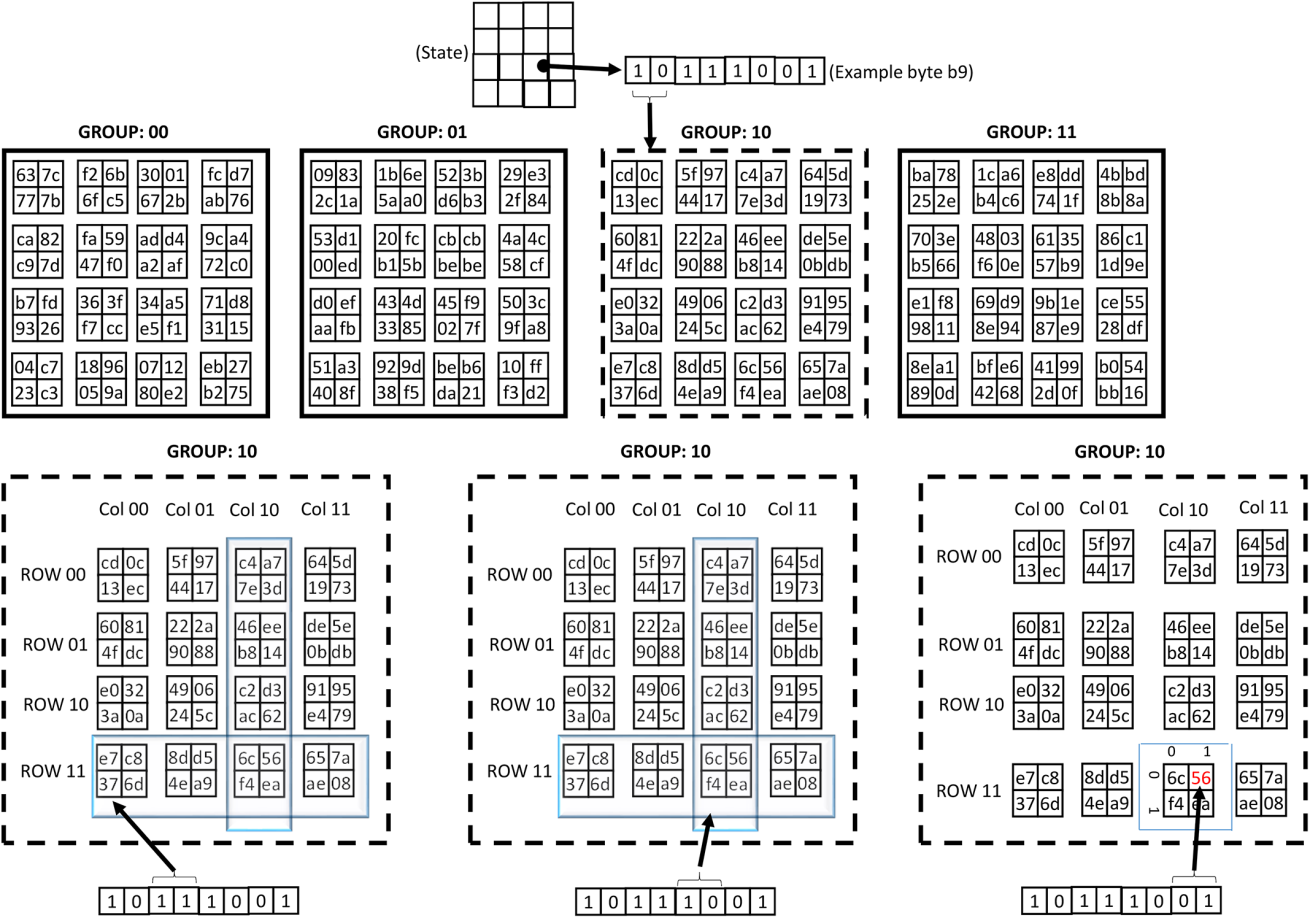
An illustrative example of the proposed S-box architecture.

**Fig 2:**
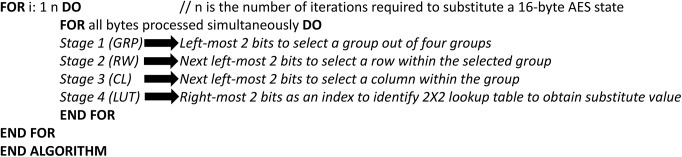
The proposed substitution byte algorithm.

To illustrate the look-up process, consider a state of 16 bytes (Fig 1). Supposedly, the byte to be substituted has the hexadecimal value of {b9}. Here binary representation of {b9} is {10111001} and is a binary string, where the most significant bit is regarded as a_7_ and the least significant bit is a_0_. The first step is group selection which is based on the a_7_ and a_6_ of the processed byte, which corresponds to Group 3 in this case. The row and column values of the corresponding group are specified by the bits a_5_-a_2_. Finally, bits a_0_ and a_1_ select the substitution value. Thus, in case of the above mentioned example, the substitution value is obtained as {56}. The steps required in the proposed substitution method are summarized in the algorithm (Fig 2).

The S-box is optimized by breaking down the large matrix into groups to eliminate the delay producing algebraic and matrix operation. Therefore, this optimization technique reduces the number of iteration to substitute a single byte which increases speed and decreases latency. As composite field design of S-box requires more arithmetic operations, it simply consumes more power compared to look up table. This proposed algorithm uses groups of small tables which is further beneficial as it simplifies table indexing and results in the reduction of delay and power consumption.

## Proposed S-box Implementation

**Fig 3:**
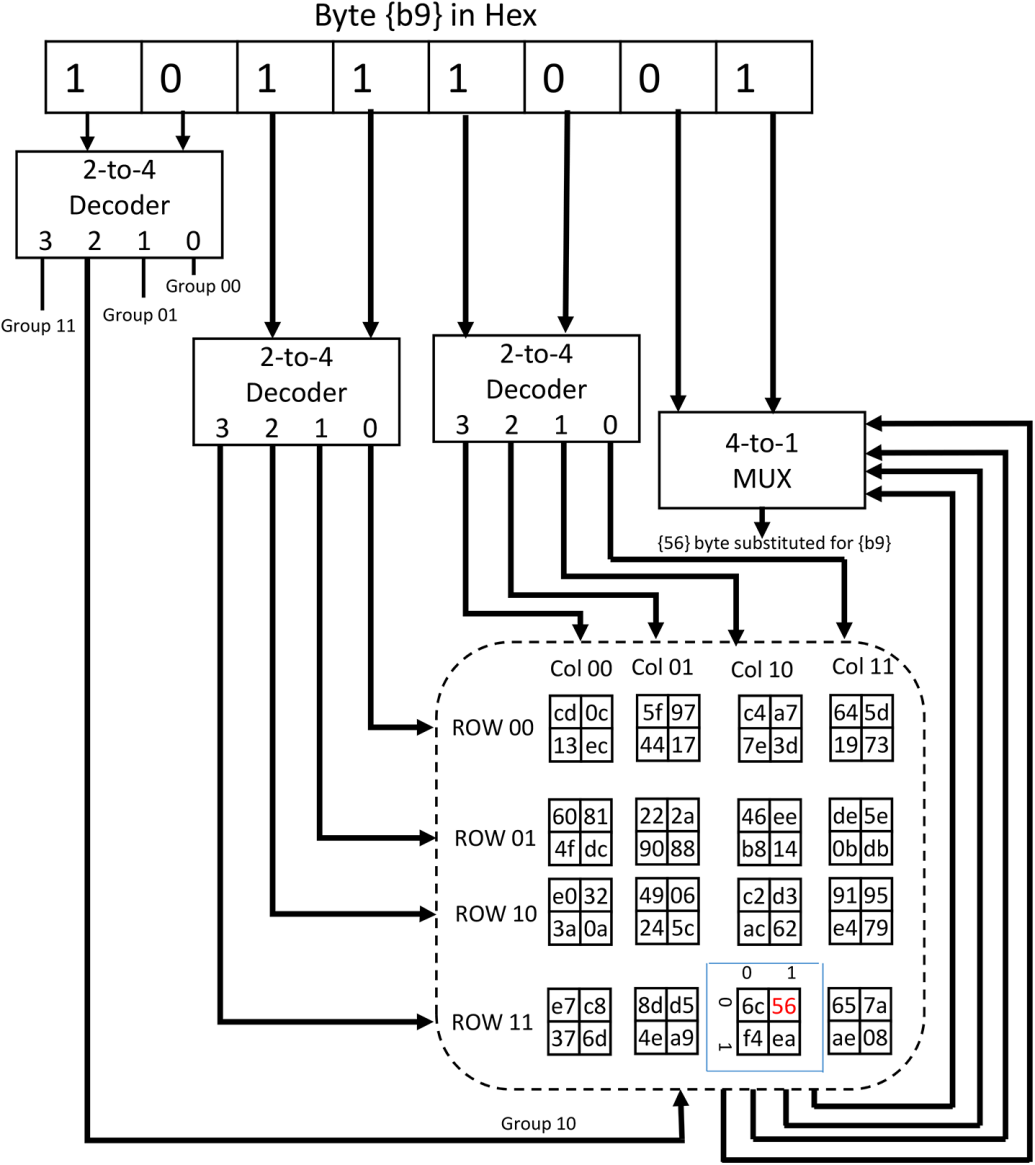
Block diagram for a single byte substitution.

This Section introduces the hardware implementation of the proposed S-box algorithm in both CMOS and FPGA. The S-box has been designed and synthesis using the 0.35 μm CMOS standard cell library with the Synopsys Design Analyzer. First, we consider the CMOS design. For each step in the algorithm (Fig 2) the requirement of the hardware components is discussed. The selection of groups, rows, and columns is implemented using decoders. On the other hand, S-box LUT is implemented using multiplexers (Fig 3).

**Fig 4:**
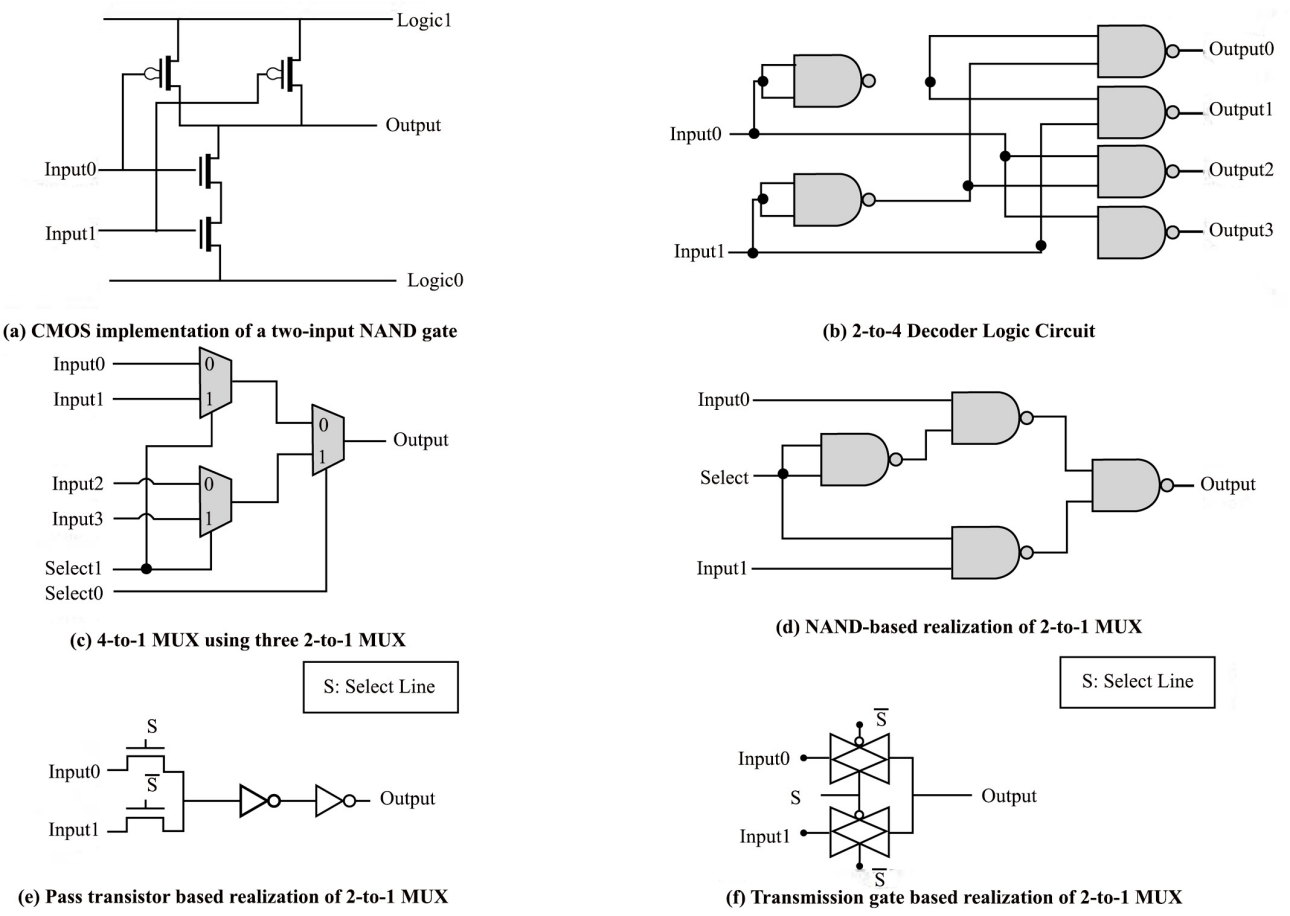
The CMOS realization circuits.

In order to choose one group out of four, a 2-to–4 decoder is used. Two more 2-to–4 decoders are required to choose the row and column within the selected group. We select to use two input NAND (NAND2) decoders to comply with the standard CMOS technology. The NAND2 and the 2-to–4 decoder circuits are shown in Fig 4(A), and Fig 4(B) respectively. Once decoding on the group, row, and column levels are done, the LUT to be used is known. The two right most bits of the byte processed are used as an index to the 2×2 LUT. These two bits are connected to the select lines of a 4-to–1 multiplexer having the table data as inputs and the S-box substitution value as the output. All the 4-to–1 multiplexers implemented are constructed using three 2-to–1 multiplexers for simplicity (Fig 4(C)). The following is an explanation of three possible designs to implement 2-to–1 multiplexers:

### First Design (Gate-Level (NAND-NAND) CMOS Implementation)

Each CMOS NAND2 gate consists of four transistors. Four NAND2 gates and an inverter can be used as shown in Fig 4(D) to perform the operation of a 2-to1 MUX. For our 2×2 LUT we need a 4-to–1 MUX which is constructed using three 2-to–1 MUX as shown in Fig 4(C).

### Second Design (Pass Transistors Implementation)

Pass transistor logic can be used as shown in Fig 4(E) to implement a 2-to–1 multiplexer. The two inverters added at the output are used to retain the logic level. The 4-to–1 multiplexer needed for S-box LUT is constructed using three 2-to–1 multiplexers.

### Third Design (Transmission Gates Implementation)

Transmission gates are simply switches which can act as two-to-one multiplexer as shown in Fig 4(F). In this case the number of transistors required is less than the former implementations discussed above. Similarly, 4-to–1 multiplexers are constructed out of 2-to–1 ones.

**Fig 5:**
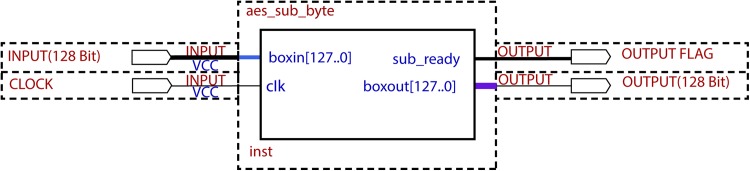
Block Diagram of S-BOX Sub module.

Second, the proposed S-box is implemented in FPGA device of family Altera, Cyclone II, EP2C35F672C6 device. The simulation is performed in Quartus-II simulation software with Verilog HDL code. In this work, 128 bit data block is given input to the S-box sub module as ‘boxin’. After getting the clock ‘clk’ the output is generated at ‘boxout’. This sub module is named as aes_sub_byte (Fig 5). The resources that have been utilized are provided in Table 1.

**Table 1:** Resource utilization in percentage for proposed s-box.

Specification	Actual quantity	Used resource	% of Utilization
Total Logic Elements	33216	57	0.11
Total Registers	3124	16	0.51
Input Output (I/O) Registers	33216	n/ a	
Total Combinational Functions	33216	57	<1
Dedicated Logic Registers	33216	16	<0.5
Total Memory bits	483840	32768	6.7
Total PLL	4	0	0

**Fig 6:**
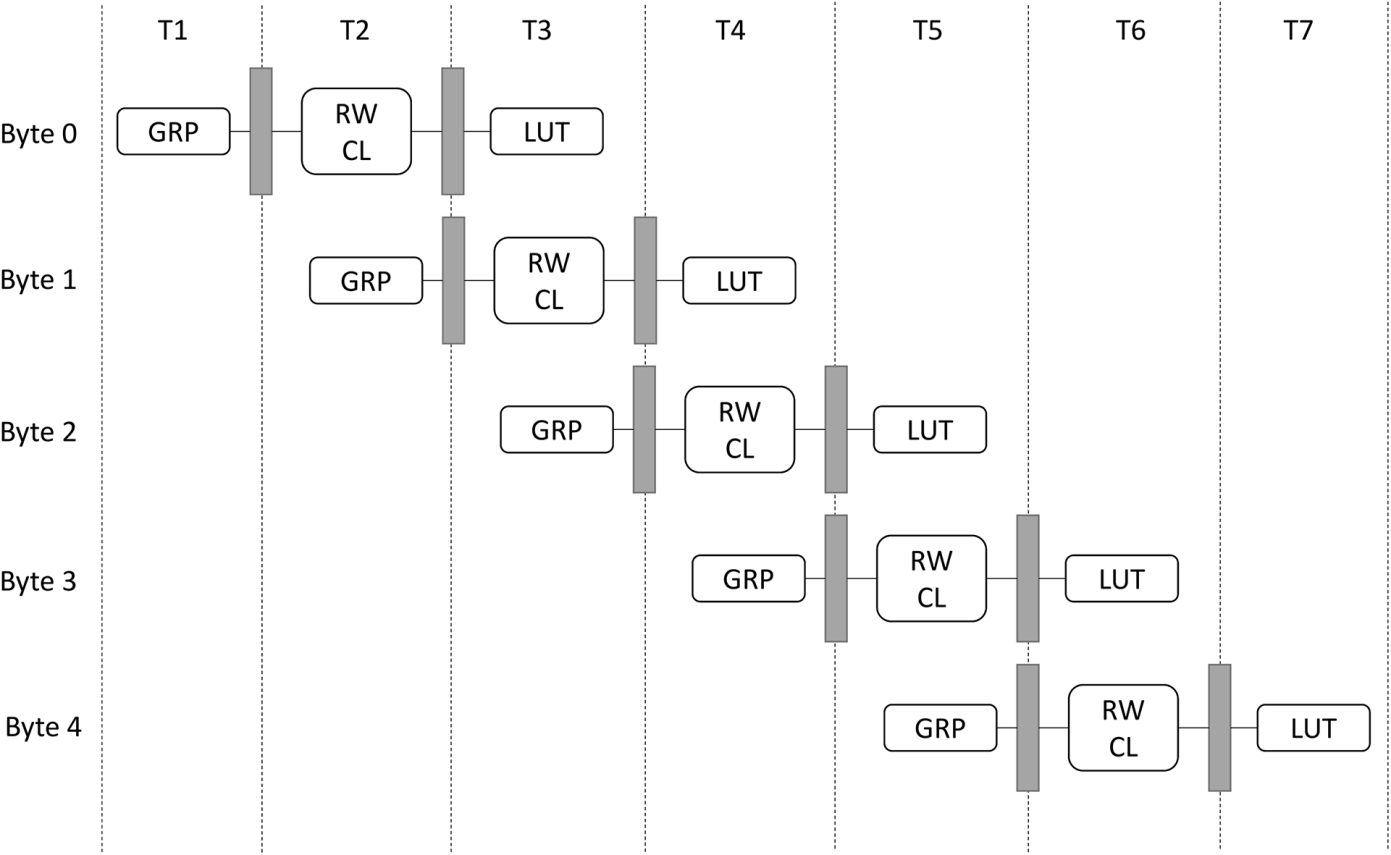
Optimized pipelined architecture for byte substitution.

The algorithm steps shown in Fig 2 can be optimized through pipelining. Each step can represent a stage in the pipeline architecture. Further speedup can be achieved by merging the second and the third steps of the algorithm since they are totally independent in terms of data and hardware resources required (Fig 6). The pipeline performance can be measured by identifying the number of stages required to complete the processing of one state substitution denoted by ‘n’, the number of iterations (m) needed to complete the processing of one state substitution, and the time (t) of the slowest stage in the pipeline.

Pipelining speed, throughput, and efficiency can be computed as discussed in [31] using Eqs 1, 2, and 3. The benefits of pipelining byte substitution can be clearly noticed as the number of bytes processed per iteration decreases. As there are no additional gates are needed when pipelined, therefore, no hardware complexities and glitches. Therefore, our proposed algorithm has low power, higher throughput and higher efficiency compare to Bertoni [23] as he used additional one-hot encoder to substitute bytes.

$$\text{Speed},\;S(n)=\frac{m\;\times n}{n+m-1}$$ (1)

$$\text{Throughput},\;U(n)=\frac{m}{(\;n+m-1)\times t}$$ (2)

$$\text{Efficiency},\;E(n)=\frac{m}{n+m-1}$$ (3)

## Results and Performance Analysis

The performance analysis of the proposed and simulated design is on the 0.35 μm CMOS technology of AMS Corporation [32]. The proposed architecture consists of two parts: the decoder unit which performs the selection among the groups and the row as well as column selection; the multiplexer unit which substitutes the value of the AES state with the S-box one. All of the three proposed designs are same for the first unit (decoder) as it is implemented with 2-input NAND gates. Therefore, the power dissipation, associated delay and area are consequently identical for the decoder part. On the other hand, all three proposed techniques share the same idea of creating a 4-to–1 multiplexer by using only 2-to–1 multiplexers. Thus, four bytes of a state require twelve 2-to–1 (Fig 4(B)), critical path delay for 4-to–1 multiplexer is twice the delay of a 2-to–1 multiplexer. Given that every four bytes of a state are processed simultaneously, the total delay is eight times that of a 2-to–1 multiplexer. With a 16-byte state, the architecture flexibility allows varying the bytes processed from a single byte at a time to 16 bytes in parallel with power of twos increments, i.e., 1, 2, 4, 8, and 16. The delay and area estimation for 1, 4 and 16 combinations are shown in Table 2. It can be observed that the more bytes processed in parallel, the more area and power are needed and the less delay is required. To clarify the results obtained, the case of processing four bytes in parallel is considered here (without pipelining).

**Table 2:** Delay, Power and Area Estimation of the Proposed Designs.

Architectural Realization	Design–1			Design–2			Design–3		
	1-byte	4-byte	16-byte	1-byte	4-byte	16-byte	1-byte	4-byte	16-byte
Decoders Delay (ns)	6.4	1.6	0.4	6.4	1.6	0.4	6.4	1.6	0.4
Multiplexers Delay (ns)	9.6	2.4	0.6	8	2	0.5	3.2	0.8	0.2
**Total Delay (ns)**	**16**	**4**	**1**	**14.4**	**3.6**	**0.9**	**9.6**	**2.4**	**0.6**
**Total Power (mW)**	**0.12**	**0.5**	**2**	**0.14**	**0.55**	**2.22**	**0.21**	**0.83**	**3.33**
Decoders Area (GE)	18	72	288	18	72	288	18	72	288
Multiplexers Area (GE)	96	384	1536	84	336	1344	48	192	768
**Total Area (GE)**	**114**	**456**	**1824**	**102**	**408**	**1632**	**66**	**264**	**1056**

**Fig 7:**
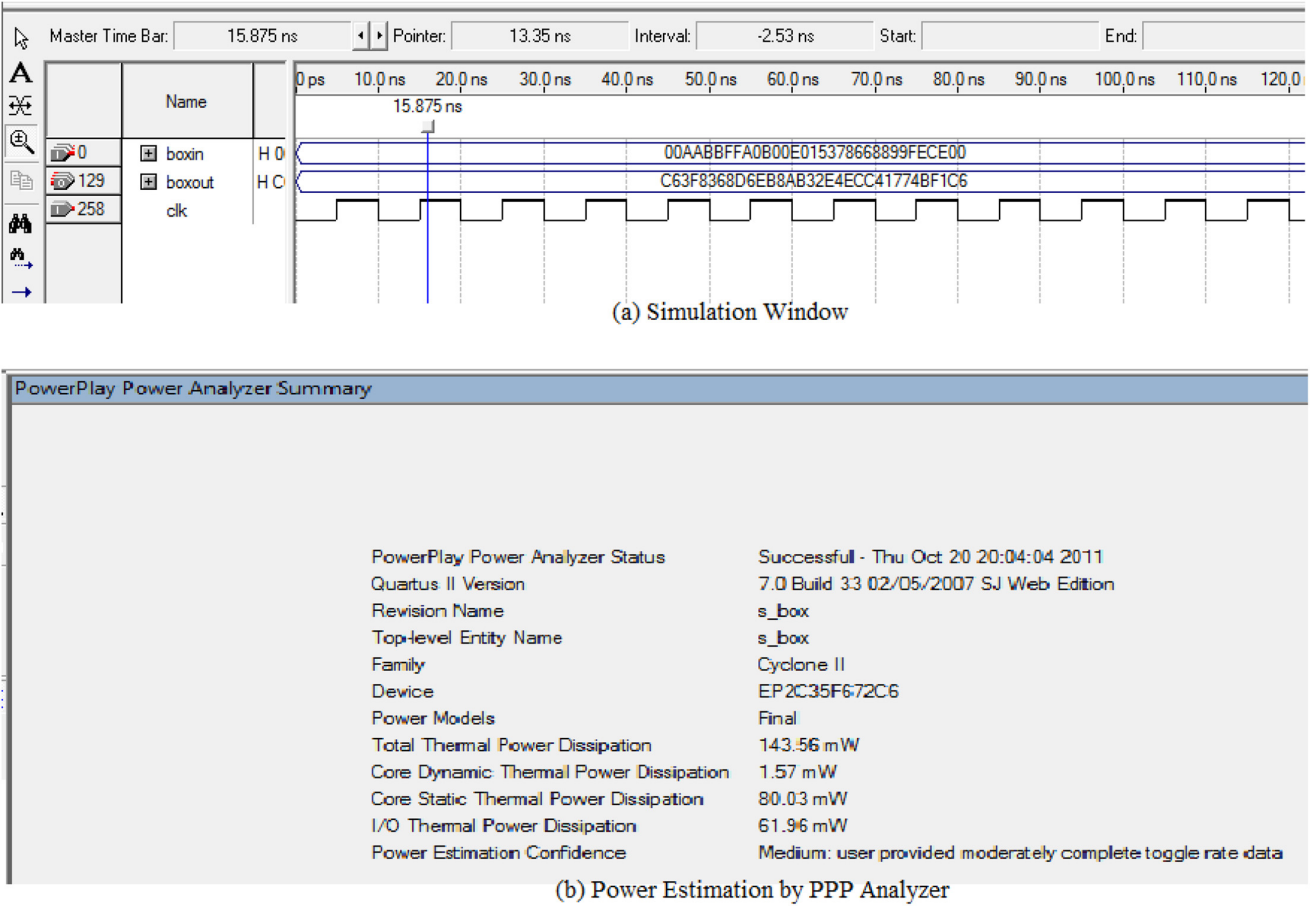
Quartus-II simulator output for power analysis of the S-box.

In the FPGA analysis, the S-box simulation is performed by Quartus-II simulator. Fig 7(A) shows the result for S-box operation. Among all the three proposed architectures the simulation result that is provided here is the third one. Here the boxin takes input as the positive edge clk getting signal and generate the 128 bit output to the boxout. All the 16-byte (128 bits) of AES state is replaced by the S-box values. The Throughput = 128 bits/ (cycle per substitution * time period) and the Latency = Clock cycles/Fclk. The timing analysis provides the maximum frequency 99.79MHz and the speed performance is determined on the basis of this clock cycle. After 4 clock cycles the input flag is one. So the latency is 4. The time periods of 99.79 MHz signal = 1/99.79×10^6^ = 10 ns. Again a cycle processes a data sample of 128 bits and it requires 4 cycles to provide output. So the throughput of the proposed processor is = 128/4*10ns = 3.2 Gbps. The Power Play Power Analyzer tool of Quartus-II simulator estimates the total power consumption of the proposed design, which is shown in Fig 7(B). Total dynamic power dissipation for S-box is obtained 1.57 mW.

Furthermore, the pipelined structure (Fig 6) that has been described by the Eqs 1, 2, and 3 is iterated in Table 3. The benefits of pipelining byte substitution can be clearly noticed, as the number of bytes processed per iteration decreases. The optimal speed and hence higher efficiency is achieved when the state is taken single byte at a time. In this particular case, the pipelined solution is 8/3 ≈ 2.67 times faster than the non-pipelined one. This is significantly fast, for one state completes its byte substitution in 6 ns rather than 16 ns for the 1-byte case. Regardless of the design selected, the intermediate cases (i.e., when 2-bytes, 4-bytes, or 8-bytes are processed per pipeline stage) represent a compromise between delay and area. Those intermediate cases take advantage of both pipelining and parallelism to reduce delay, while consuming reasonable hardware resources.

**Table 3:** Pipeline architecture evaluation.

Comparison Criterion	Design–1			Design–2			Design–3		
	1-byte	4-byte	16-byte	1-byte	4-byte	16-byte	1-byte	4-byte	16-byte
Number of Iterations, m	16	4	1	16	4	1	16	4	1
Stage 1: Group decoding delay(ns)	3.2	0.8	0.2	3.2	0.8	0.2	3.2	0.8	0.2
Stage 2: Row/Col decoding delay(ns)	3.2	0.8	0.2	3.2	0.8	0.2	3.3	0.8	0.2
Stage 3: Multiplexers delay(ns)	9.6	2.4	0.6	8	2	0.5	3.2	0.8	0.2
Maximum stage delay normalized, t	48	12	3	40	10	2.5	16	4	1
Speed, S(n)	2.67	2	1	2.67	2	1	2.67	2	1
Throughput, T(n)	0.02	0.07	0.3	0.02	0.09	0.36	0.06	0.22	0.89
Efficiency, E(n)	0.89	0.67	0.33	0.89	0.67	0.33	0.89	0.67	0.33
**Total delay (ns)**	**16**	**4**	**1**	**14.4**	**3.6**	**0.9**	**9.6**	**2.4**	**0.6**
**Total delay with pipelining (ns)**	**5.99**	**2**	**1**	**5.39**	**1.8**	**0.9**	**3.6**	**1.2**	**0.6**

The performance of all the three designs, with and without pipelined, are explained in this Sections and the results are listed in Table 4 in the next Section. In this table we compare our work with other recent related works in terms of power, area, area-power product and area-delay squared product with respect to target critical path delay. The next Section will show these comparisons in graphs.

## Comparison

In this Section, we list all the proposed designs including pipelined design alongside other related works (Table 4). A comparison of the proposed designs with the state of the art substitution box implementations have been shown graphically. Delay and area values for the existing techniques are obtained from the survey done by Tillich et al [24]. As stated earlier our design is implemented as a combination of hardware look-up table and calculation of S-box, we simply compare our architecture with other recent literatures.

**Table 4:** Delay, Power and Area Comparisons.

Implementation		Area, *A* (GE)	Critical Path Delay, *τ* (ns)	Area Delay Squared Product Normalized, *Aτ* ^*2*^	Power Consumption (mW)	Area, Power Product Normalized
	Wolkerstorfer [5]	382.5	8	0.800	1.1	420.75
	Wong [18]	95	2.5	0.02	34.98	3323.1
	Bertoni [23]	1608.75	3	0.473	1.86	2992.3
	hw_lut [24]	1203.75	3	0.354	2.5	3009.4
	Tillich [24]	1912.5	4	1.000	1.57	3002.6
	Satoh [26]	360	9	0.953	30.55	10998
	Canright [27]	281.25	8	0.588	28.44	7999
	Elazm [28]	415.3	6	0.49	18.62	7732.8
	Shanthini [29]	99	6.275	0.127	503.9	49886.1
	Li [33]	1920	2.8	0.492	n/a	n/a
	Nabihah[34]	147	3.235	0.05	7.98	1173
	Hongge[36](FPGA)	28BRAM+ 544 LE	97	n/a	0.575	328.9
	**Our Design (FPGA)**	**57 Reg+16 LE**	**40**	**n/a**	**1.57**	**114.6**
**First**	1-byte	114	5.99	0.954	0.12	14
**Proposed**	4-byt e	456	2	0.238	0.5	228
**Solution**	16-byte	1824	1	0.060	2	3648
**Second**	1-byte	102	5.39	0.691	0.14	14
**Proposed**	4-byte	408	1.8	0.173	0.55	224.4
**Solution**	16-byte	1632	0.9	0.043	2.22	3624
**Third**	1-byte	66	3.6	0.199	0.21	14
**Proposed**	4-byte	264	1.2	0.050	0.83	219.12
**Solution**	16-byte	1056	0.6	0.012	3.33	3516

Note: normalization constant, X is found by the equation such as; X×V^2^×f = P, where p is the given power. Our delay data is pipelined.

**Fig 8:**
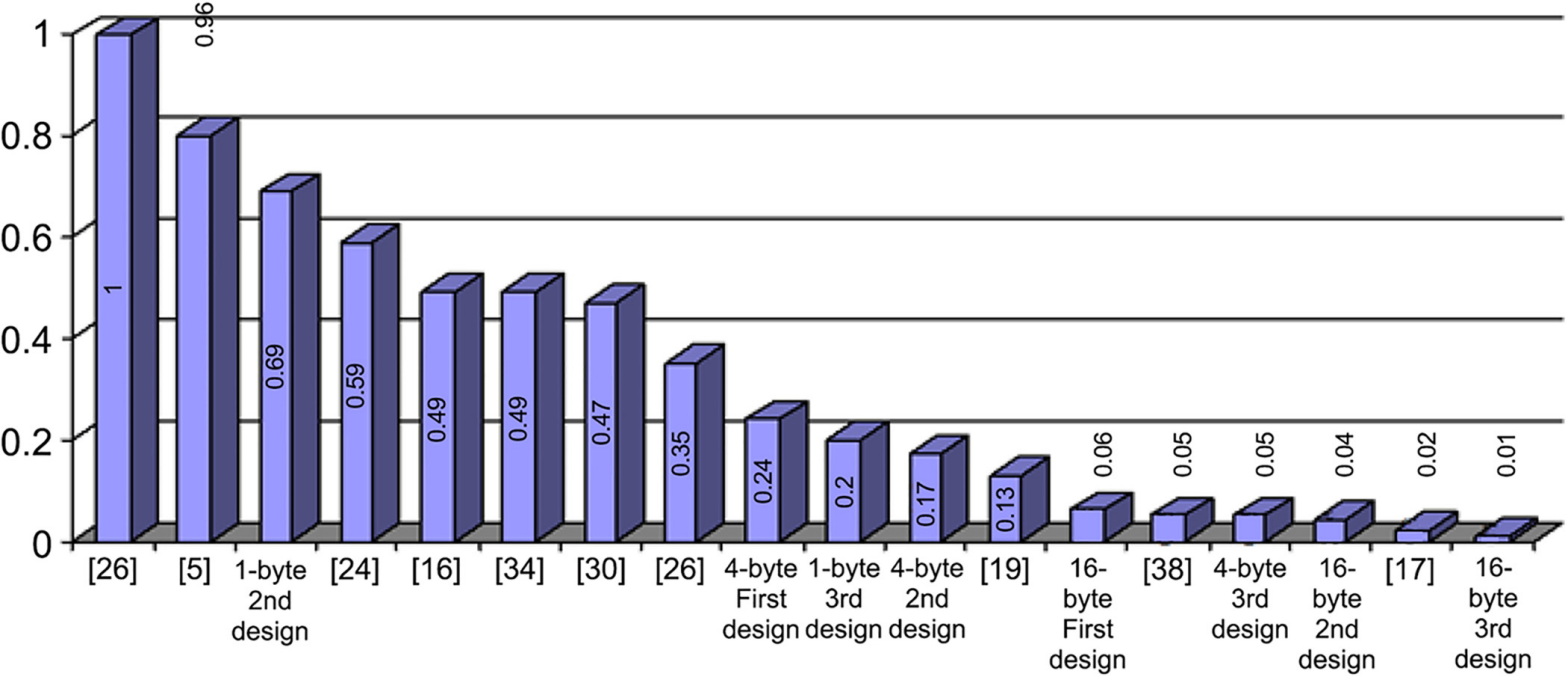
Area, delay squared product of different works.

The work of Bertoni [23], Tillich [24] and Li [33] presents the hardware LUT implementations and reports a significant improvement in critical path delay along with low power at the expense of silicon area. Amongst three designs, Li’s delay is minimum (Table 4) but large in size. As he decomposes the S-box with 32 small tables, his design requires a flag bit in each table. If the flag bit is ‘on’ the controller is kept waiting which results in delay and requires more area for this extra flag bit. This proposed architecture selects a group without checking any flag bit, thus reducing the delay. The area delay squared product graph (Fig 8) shows that our design performs much better compare to Li, Tillich and Bertoni’ work.

**Fig 9:**
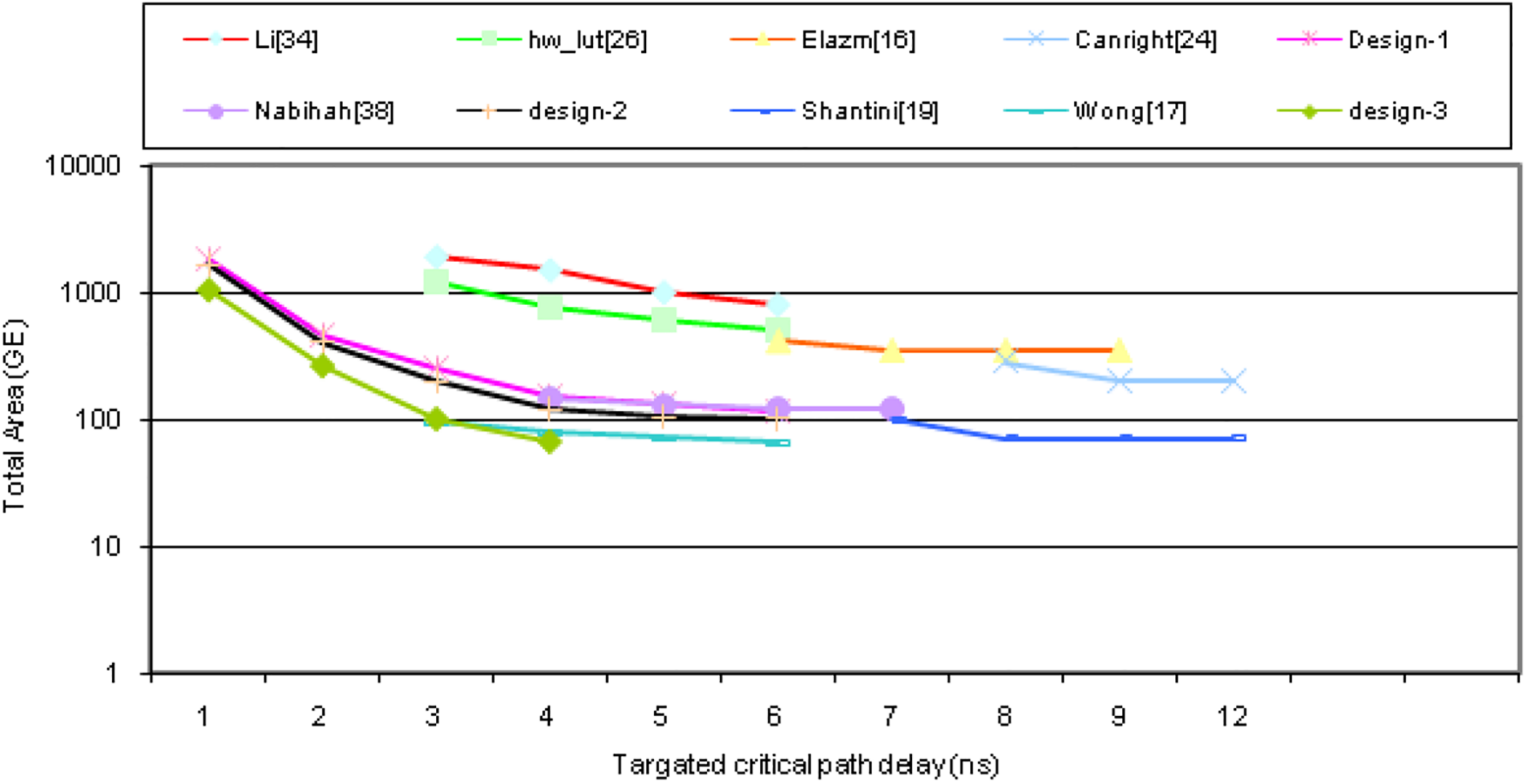
Area (GE) versus target value for critical path delay (ns).

The area-delay graph shows the better performance of our design when synthesized for a specific critical path delay (Fig 9). Each legend cites the functions in the same top–down order as they are contained in the respective Fig. The area is given in gate equivalents (GE) and calculated as total area divided by the size of a two-input NAND with the lowest drive strength (Table 2). Amongst the three implementations (at the bottom of the Fig 9), our proposed Design–3 is clearly the best. It has the smallest size out of all ten S-boxes. Though Wong’s implementation has almost same silicon area, it suffers from a longer critical path. The other four designs including Nabihah [34] and Choi [35] are the calculating implementations and show smaller area than the other three works [24, 28, 33]. This is because they make use of the algebraic structure of the S-box to implement the substitution. On the other hand, these structures have a relatively long critical path. The shortest critical path can be achieved by hw_lut [24] and Li [33], but their size is about three to four times higher in magnitude than that of Proposed Design–3. That is because the synthesizer of proposed work has a much higher degree of freedom for optimizing the circuit, which allows for a shorter critical path.

**Fig 10:**
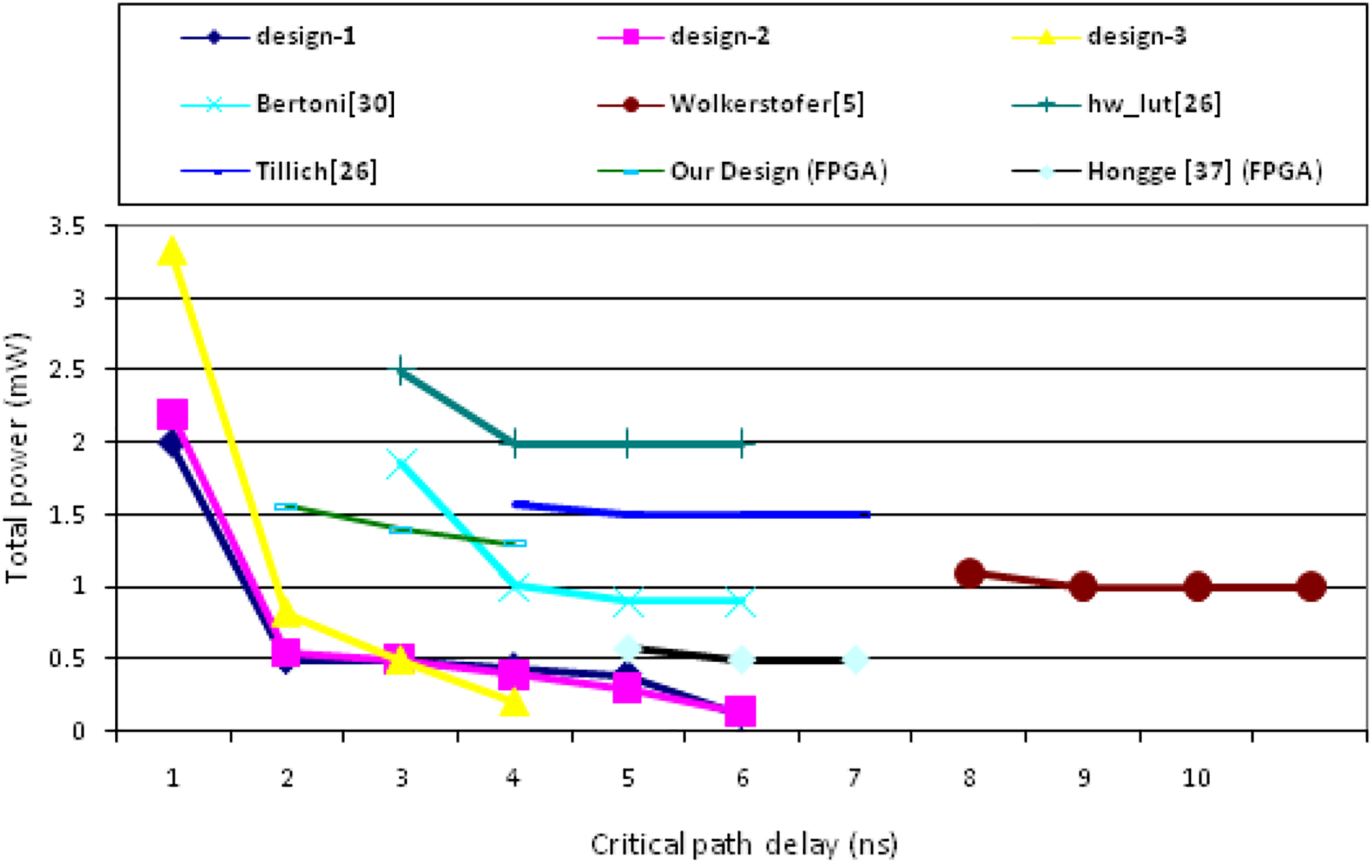
Total power vs. target critical path delay.

There are seven designs including the proposed works have been plotted (Fig 10). The proposed design of FPGA implementation is also plotted against most recent FPGA implemented design of Hongge [36]. The FPGA implementation results much higher delay compare to CMOS implementation. Therefore, the delay is normalized by a factor of twenty. It is clearly shown that the power consumption of Hongge’s design consumes much less power than the proposed design. This is because the reconfigurable architectures in cell array reduce the glitches in the routing paths. However, the critical path delay is more than twice that obtained in the proposed design. The reconfiguration cell array processing elements are connected to each other through the interconnect routing and switchbox in Hongge’s design. The operation of SubBytes and XOR is implemented with internal cell of array. Due to the interconnected routing and more switching it has long delay and large area.

Moreover, all the proposed design outperformed the literatures [5, 23, 24]. A very close outcome is seen in Bertoni’s design with an optimum critical path delay, but, the power consumption is around twice. Wilkertofer’s s-box achieves similar results but the long critical path is a disadvantage. The other two approaches consume three times as much power as the proposed design, while hw-lut [24] consumes about four times more power.

**Fig 11:**
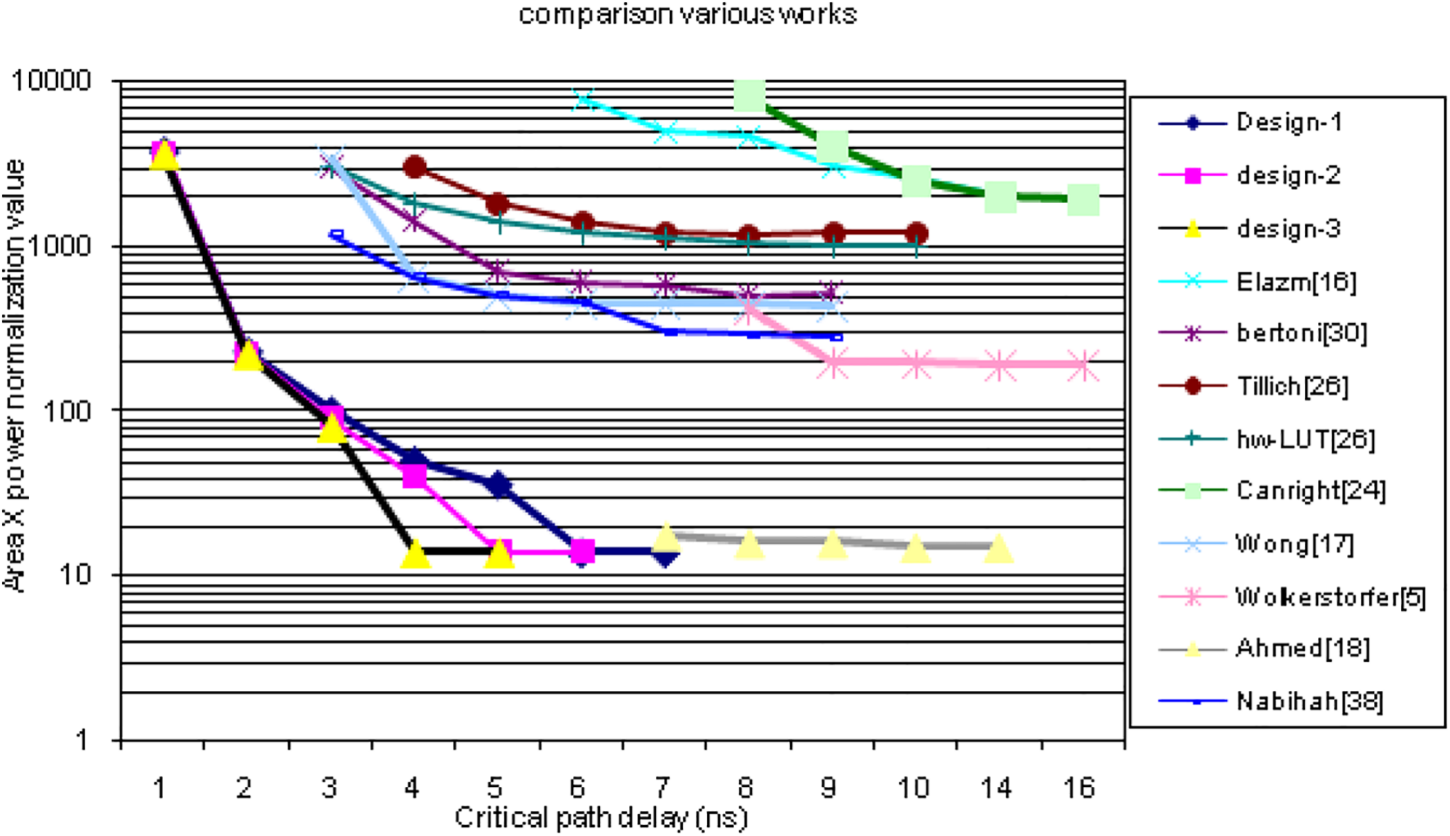
Area, power product vs. target critical path delay.

Fig 11 shows the area-power consumption graph plotted against the target critical path delay. All the literatures are not shown in the graph because the normalized outcome of some literatures is too large compared to the proposed designs. It is clearly shown that the proposed design–3 has minimum area-power product compared to the designs [5, 18, 23, 24, 27, 28, 30, 34]. The proposed design have less iteration or indexing as it has been broken down small tables. Furthermore, these ensure no extra internal flip flops in between transitions which in turn reduces the signal activities. Therefore, the signal activity within that particular path is low, which limits the overall power consumption. Amongst the eight, Wolkerster [5] shows less area power product compare to others, but suffering large critical path delay. The second better performance comes from Nabihah [34] with very good critical path delay. The area power product is slightly higher than Wolkerster’s because the power consumption of [34] is seven times higher in magnitude due to calculation of S-box in combinational basis (polynomial basis).

## Conclusion

This paper discusses the design and simulation of a new AES byte substitution technique. It is initiated and implemented in three different hardware combinations in 0.35 μm CMOS technology. The design is also simulated in the Quartus-II simulator for an FPGA platform to determine its power, delay and area. The proposed pipeline architecture of S-box shows that the throughput can be maximized by reducing the delay of the critical path. Compared to other well-known previously reported techniques, the analysis of the results indicates that the proposed design is capable of significantly outperforming the existing solutions in terms of power, delay and area as measured by simulation.
